# The effectiveness of sensory stimulation therapy to strengthen the resilience of operating room nurses

**DOI:** 10.4102/curationis.v39i1.1590

**Published:** 2016-10-26

**Authors:** Chantal Marais, Emmerentia Du Plessis, Magdalene P. Koen

**Affiliations:** 1 Theatre, Wilmedpark Hospital, South Africa; 2 School of Nursing Science, North-West University, Potchefstroom Campus, South Africa; 3 Department of Nursing, North-West University, Mafikeng Campus, South Africa

## Abstract

**Background:**

Operating room (OR) nurses need to be resilient in order to cope with extreme demands in their workplace. This research focused on the effectiveness of sensory stimulation therapy (SST) to strengthen the resilience of nurses in the OR of a private hospital in the North West Province.

**Purpose:**

The purpose was to determine the effectiveness of SST as an intervention to strengthen the resilience of OR nurses.

**Design:**

A quasi-experimental design was used.

**Method:**

The population consisted of OR nurses and ICU nurses at private hospitals in the North West Province. All-inclusive sampling was used. Forty-one OR nurses formed the intervention group. A pilot group (8 subjects, OR nurses), as well as a comparison group (23 subjects, ICU nurses), was also sampled. An intervention, namely SST, was implemented with the intervention group. The resilience of the intervention group, pilot group and comparison group was measured before and after the implementation of the SST by means of Wagnild and Young’s resilience questionnaire. The intervention group also completed a self-report questionnaire on their needs and suggestions for SST and wrote short narratives on their experience of SST. Data were analysed using descriptive and inferential statistics, and by thematic coding.

**Results:**

Results indicated a significant statistical increase in the intervention group’s resilience levels. Results from the narratives confirmed that the intervention group’s resilience may have been strengthened through SST.

**Conclusion:**

SST has potential to strengthen the resilience of OR nurses.

## Introduction and background

This research focused on the effectiveness of a sensory stimulation therapy (SST) intervention to strengthen the resilience of nurses in the operating room (OR) of a private hospital in the North West Province.

Nurses in general report stress predominantly related to the work environment and burnout (Vowels, Topp & Berger 2012) because of high health-care risks and increased workload, in terms of infectious diseases, confrontations with death and suffering, poor communication and social support, shift work and emotional demands (Firth-Cozens 2001; Pisanti *et al.* 2003). Contributing factors to stress also include the high number of inexperienced nurses (Gillespie *et al.* 2007), stress of working with too many patients (Hegney *et al.* 2006) and spending insufficient time with patients in need (Boykin *et al.* 2003).

In addition, making life-changing decisions in limited time, continuous contact with other members of the multi-professional team who are also severely stressed, and conflict situations with colleagues also contribute to stress (Vowels *et al.* 2012). Staff shortages and excessive administrative duties are also rated as severely stressful by nurses (Van der Colff & Rothmann 2009). The well-being of nurses experiencing high levels of stress is thus at risk, especially in highly specialised departments, such as intensive care units and ORs.

Focusing on the OR, these units can be defined as specialised departments in the hospital. Specialised nursing areas are complex departments, especially because of the unique stressors associated with those specific areas. Physician abuse, poor communication, overlapping responsibilities, ethical problems, perceived aggressive behaviour between OR personnel, emotional labour and teamwork problems are unique stressors in the OR (Coe & Gould 2008; Higgins & MacIntosh 2010). These problems lead to job dissatisfaction, a decrease in the well-being of nurses and insufficient patient care (Näslund Andréasson 2011; Woldehawariat 2012). Thus, OR nurses are exposed to higher levels of stress which can influence their own well-being negatively (Vowels *et al.* 2012).

OR can indeed be identified as one of the most stressful departments in health care (Niasar *et al.* 2013). From the researcher’s own experience as a professional trained theatre nurse, shortages in experienced nurses and a stressful work environment do have an influence on effective safe patient care. Because of inadequately skilled theatre nurses, highly skilled and qualified nurses have to take responsibility for some of the unskilled theatre nurses workload, resulting in impeded patient safety. High stress levels, burnout, sleep deprivation, bad working relationships and a high nursing staff turnover are some of the negative effects in OR as a result of these extra responsibilities (Higgins & MacIntosh 2010; Niasar *et al.* 2013).

The current situation of OR nurses emphasises the need for a positive working environment. According to Rondeau and Francescutti (2005), a positive working environment can be defined as a setting that supports excellence and that facilitates the resilience of staff. Resilience might indeed be the factor that contributes to the fact that some OR nurses choose to remain in the OR in spite of difficulties (Koen, van Eeden & Wissing 2011a). Thus, there is a need for a deeper insight and understanding of the resilience of OR nurses, and to explore strategies to strengthen resilience in the working environment of OR nurses. One strategy to strengthen the resilience of nurses may be SST, which may also contribute to a positive work environment (Koen & Du Plessis 2011).

### Problem statement

The well-being, including the resilience, of OR nurses might be at risk, leading to high staff turnover and possible low standards of patient care as a result of their adverse working conditions (Woldehawariat 2012). A comprehensive approach is necessary to contribute to a more positive work environment for OR nurses (Näslund Andréasson 2011; Registered Nurses Association of Ontario 2006) and to strengthen OR nurses’ resilience (Koen & Du Plessis 2011). As part of a research programme to strengthen the resilience of health caregivers – the RISE programme – Koen and Du Plessis (2011) suggest that it might be valuable to explore and describe SST as an intervention to strengthen nurses’ resilience.

OR nurses in a private hospital in the North West Province seemed to experience a very low morale and decreased productivity because of shortages of experienced OR nurses and a high workload. These OR nurses need an opportunity to develop resilient attributes in order to ultimately improve quality in health care. It was not clear if SST might be effective to strengthen the resilience of nurses, leading to the following research question: Can the resilience of OR nurses be strengthened by means of SST?

#### Purpose and hypothesis

The purpose of the study was to determine the effectiveness of SST as an intervention to strengthen the resilience of OR nurses in a private hospital in the North West Province.

The following hypothesis was formulated:

H1: Participating in SST will strengthen the resilience of OR nurses in a private hospital in the North West Province

This research formed part of a research programme, namely the RISE study (Koen & Du Plessis 2011). The purpose of RISE is to develop a comprehensive, multi-faceted approach to strengthen the resilience of health caregivers, including professional nurses, as well as risk groups. In this case, the focus was on OR nurses.

#### Research objectives

The following objectives were formulated:

1. To explore and describe OR nurses’ needs regarding SST in a private hospital in the North West Province.
2. To explore and describe OR nurses’ suggestions regarding the implementation of an SST in an OR environment in a private hospital in the North West Province.
3. To determine the effectiveness of an SST intervention to strengthen the resilience of OR nurses in a private hospital in the North West Province.

#### Definition of key concepts

**Resilience**: Resilience is an adaptive quality in the presence of adversity, contributing to independent functioning and well-being (Aroian & Norris 2002). Nurses in the OR might experience workplace adversity, and they need resilience to cope.

**Operating room**: An OR is a restricted area where surgery takes place (Phillips 2013). It includes operating theatres, recovery rooms, stores, areas where staff prepare for surgery and a pre- and post-operational care section.

**Operating room nurse**: For the purpose of this research, OR nurse or nurse refers to a peri-anaesthesia nurse, the circulating nurse, the scrub nurse and the nurse as part of the non-sterile team. These nurses may be professionally trained theatre nurses, professional nurses, enrolled staff nurses or auxiliary nurses.

**Sensory stimulation therapy**: SST is a combination of sights, sounds, textures, aromas and motion that stimulates the primary senses (Collier *et al.* 2010). The purpose of SST is simultaneous stimulation and relaxation without any intellectual input or mastery of skills required. In this research, SST entailed an intervention designed by the researcher, based on literature and guided by the research supervisors and the feedback obtained from subjects.

#### Contribution to the field

This research explores SST as a possible intervention to strengthen the resilience of OR nurses. This might be valuable to OR health service managers to consider SST as a tool to create a work environment in which OR nurses’ resilience might be strengthened.

### Literature review

A literature review was conducted to explore concepts related to this research. Databases and search engines were used, namely Science Direct, Sabinet, PubMed and EbscoHost, using the following key words: well-being, resilience, OR nurses and SST.

#### Resilience and OR nurses

The OR is a fast-paced setting and OR nurses are expected to work at a quick pace and with precision to ensure optimal patient safety. Their resilience and ability to cope in the OR is thus of utmost importance. OR nurses thus need attributes such as competence, self-esteem, continual growth and flexibility, coping, self-help, communication and problem solving skills (Garmezy 1991). Professional nurses with higher levels of resilience do reflect such characteristics, namely hope, optimism, coping, self-efficacy, sense of coherence, mental health and overall well-being (Koen *et al.* 2011b).

Looking at resilience specifically, this concept can be described as a dynamic process in which the individual positively adapts to adversity or risk (Friedli 2009; Herrman, Saxena & Moodie 2005; Masten & Reed 2005). After a stressful event, the resilient individual has the capacity to rebound and attain a healthy outcome (Rutter 2007; Silver 2009). Resilience can be eminent within persons (coping and optimism), among persons (social support) and across social levels (educational systems) (Masten & Reed 2005). Furthermore, resilience has five imperative characteristics, namely purpose, perseverance, self-reliance, equanimity and existential aloneness (Wagnild 2010).

#### Sensory stimulation therapy

SST is practised as multisensory rooms and is an intervention developed to improve the quality of life of persons through gradual introduction to pleasurable sensory experiences within an atmosphere of trust and relaxation. The primary senses (see, hear, smell, feel and taste) are gently stimulated without the expectation of intellectual activity (Collier *et al.* 2010). Instructions are limited and the person has control and choice to use the multisensory room according to his or her preference. SST is created according to individual needs, in any suitable area by any member of the multi-professional team (Baillon, Van Diepen & Prettyman 2002; Bera 2008).

SST is widely implemented for stress management and relaxation, in the care of intellectually disabled individuals, in the management of chronic pain, in mother and child care and in dementia care (Collier *et al.* 2010; Van Weert *et al.* 2006). The use of this therapy may make a significant contribution to the resilience of OR nurses as it might give them the opportunity to relax and recover from stressful events.

Available research on SST mainly focuses on intellectually disabled persons and persons with dementia (Asher *et al.* 2010; Fava & Strauss 2010) as well as the neurology associated with SST (Dang-Vu *et al.* 2010; Korosi & Baram 2009), whilst SST for OR nurses in a private hospital in the North West Province seems to be unexplored.

## Research method and design

### Design

As no research has previously been conducted regarding the implementation of SST in an OR as part of an approach to strengthen resilience a quasi-experimental design (Polit & Beck 2012) was used. The researcher decided on the specific design for the following reasons: A small sample size was available for the study. Thus, non-randomisation was applied. Furthermore, a natural environment was used for the study so that the findings can be generalised to similar departments in the hospital environment. Although the sample size was small, the findings may be generalised to specific related departments in the hospital. The intervention was implemented over a time period of 2 consecutive months. A pre- and post-test contributed to accurate results after the implementation of the intervention.

### Method

#### Population

This research was conducted in private hospitals in an urban setting in the North West Province. The OR department of a private hospital was included and consisted of OR theatres, a central sterilisation department and recovery rooms. An ICU in the same hospital and an OR department of another close-by private hospital was included for the purpose of a comparison group.

The population included professionally trained theatre nurses, professional nurses, enrolled staff nurses and auxiliary nurses working in the OR of two private hospitals in the North West Province. For the purpose of a comparison group ICU nurses were included, as they experience a highly similar fast-paced and challenging work environment.

#### Sample

All-inclusive sampling of nurses working in the OR and ICU departments was used. To recruit subjects, the first author presented the intended research project to the management of the two private hospitals as well as to all nurses of different departments in order to obtain permission. Before collecting the data the researcher held information sessions to inform potential subjects about the planned study, including the research problem, the purpose and objectives of the study, the data collection methods, SST and ethical considerations.

Written consent was obtained from the management of the two private hospitals as well as from subjects in the research. Subjects had the right to self-determination in the sense that they could withdraw from the research at any time without penalty. They participated voluntary, and written consent was obtained.

**Intervention group**: The intervention group included professionally trained theatre nurses, professional nurses, enrolled staff nurses and auxiliary nurses working in the OR of a private hospital in the North West Province. An all-inclusive sample of 41 OR nurses was taken.

**Pilot and comparison groups**: Pilot group and comparison group were also sampled. These groups had characteristics highly similar to that of the population, namely that they were nurses working in a specialised, complex and stressful work environment. The pilot group included an all-inclusive sample of eight (*n* = 8) OR nurses from another private hospital than the intervention group. The comparison group included an all-inclusive sample of 23 (*n* = 23) ICU nurses. The intervention group and the comparison group were situated in the same private hospital. The comparison group did not undergo the intervention, namely SST.

### Data collection

Using a pilot, intervention and a comparison groups, pre- and post-intervention data were collected from all three groups to evaluate the impact of the SST intervention and to rule out internal validity threats (Christensen, Johnson & Turner 2011; Polit& Beck 2012).

**Pilot study**: A pilot study was conducted with 8 subjects in a smaller OR in a private hospital to ensure that instructions and the resilience questionnaire were clear and to determine unanticipated effects (Polit & Beck 2012). The subjects indicated that they found the questionnaire clear and they did not suggest any changes. Thus, the resilience scale questionnaire was deemed feasible, reliable, valid and useable in this specific population. The data collected by means of the pilot study could be included as comparison group data, as it was highly similar to data collected from the actual comparison group.

**Pretest**: All three participating groups completed a resilience questionnaire developed by Wagnild and Young (1993) as the pretest to determine their resilience levels. The resilience questionnaire has been validated in previous studies (Girtler *et al.* 2010; Nishi *et al.* 2010; Wagnild 2009; Wagnild & Young 1993), and permission to use the questionnaire was obtained from the original authors.

Resilience has five imperative characteristics on which Wagnild and Young developed the resilience scale, namely purpose, perseverance, self-reliance, equanimity and existential aloneness (Wagnild 2010). Questions are scored on a 7-point scale, ranging from 1 = disagree to 7 = agree. Final scores on the resilience scale can vary between 25 and 175. The higher the score, the more resilient an individual is. The following are various resilience levels: 25–100 very low, 101–115 low, 116–130 moderate low, 131–145 moderate high, 145–160 high and 161–175 very high (Wagnild & Young1993).

The scale represents a high degree of internal consistency (Abiola & Udofia 2011). During the pretest in this research, a good internal consistency with a Cronbach’s alpha coefficient of 0.88 was also obtained.

In addition, a structured self-report questionnaire was completed by the intervention group only, to determine their need for SST and suggestions regarding SST. The self-report questionnaire was compiled by the researcher, based on the objectives of the research and with the guidance of research supervisors. This questionnaire contained open and closed-ended questions with regard to subjects’ pre-knowledge of SST, previous participation in SST, their need for SST in the OR and their suggestions with regard to SST in the OR. These suggestions were taken into consideration when developing the SST intervention.

**FIGURE 1:**
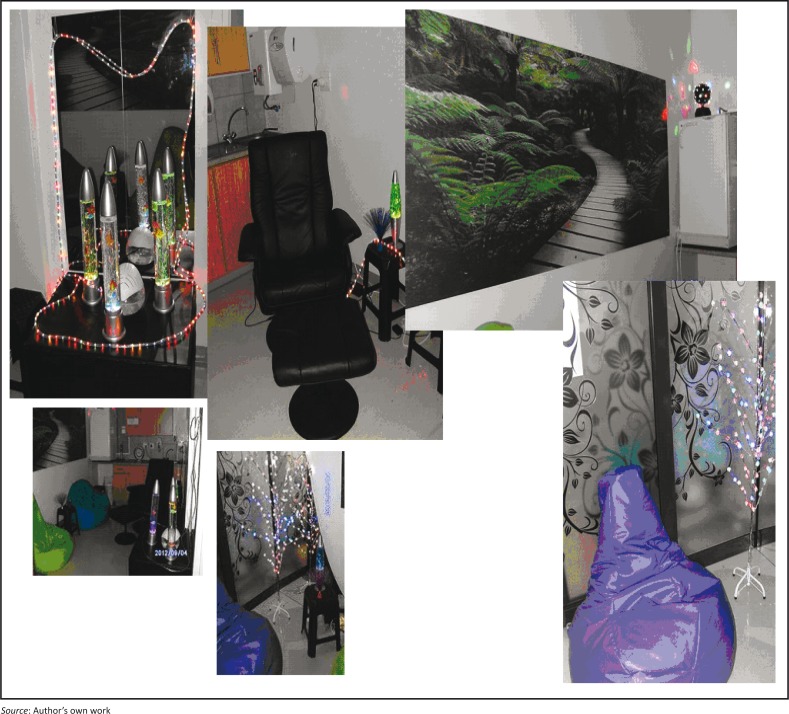
Sensory stimulation room in the operating room (OR).

**SST intervention**: An SST intervention, namely a sensory stimulation room, was put in place and implemented with the intervention group for a period of 2 consecutive months. The sensory stimulation room was designed and implemented in the OR where the intervention group was situated, based on the intervention group’s needs and suggestions as well as according to relevant literature on SST. The sensory stimulation room was equipped with the following stimulating equipment: Bean bags and a massage chair to stimulate tactile senses, bubble tubes, laser optics, fibre optics and a disco ball continuously reflecting against the walls and mirrors stimulating individuals’ visual and tactile senses. It also included a nature wall unit with calm and relaxing effects to stimulate visual senses; aroma therapy with different aromas to stimulate the olfactory senses and soft, calming background music stimulating the auditory senses. Coffee, tea and sweet savouries were provided occasionally to stimulate subject’s gustatory senses. Dim lighting in the room had a relaxed and calm effect. Figure 1 illustrates the SST used in this study.

The use of the SST room was monitored by means of an anonymous attendance list. Each subject reported their visit to the SST room by writing the date and time spent in the room using an anonymous code on the attendance list. Subjects’ codes were only known by themselves and the researcher. A total of 396 visits were recorded during the 2 consecutive months. The most visits by one subject were 25 times and the least visits were 0 because of resignation and sinus reactions caused by the aroma therapy. The longest time spent in the SST room was approximately 30 min.

**Post-test**: After the implementation of the SST intervention, post-test data were collected from the intervention and comparison groups. Both groups once again completed the resilience questionnaire. The intervention group was also requested to write a short narrative on their experience in the SST room, in order to enrich results.

### Data analysis

Descriptive inferential statistics were appropriate methods to analyse the gathered data and included measures such as frequency distribution, central tendency, variability and measures of relationships (Kremelberg 2011).

ANOVA theory was used with inferential statistics (Kremelberg 2011). Before the intervention, the different resilience levels of the groups were determined by ANOVA. ANOVA compares the statistical significance between two or more groups (Christensen *et al.* 2011).

A dependent *t*-test applied to each group in order to compare the method between the groups before and after the intervention. If *p* > 0.05 there will be no significant change. If *p* < 0.05, there will be a definite change.

To compare the method between the groups after the intervention an ANCOVA test was used where results were controlled for pre-resilience scores.

A statistical consultant assisted the researcher with the analyses of the data by means of STATISTICA (version 10) and SPSS (version 20, released 20.0.0) programmes (SPSS 2011; StatSoft 2011).

The self-report questionnaire and narratives were analysed by means of thematic coding (Howitt & Cramer 2010).

## Results

### Previous experience of SST; need for SST and suggestions regarding SST

The results of the self-report questionnaire were that subjects of the intervention group all indicated that they have never participated in an SST intervention before and it was a first-time experience for them. All the subjects identified the same area for implementation of the SST room in the OR, namely a tea room. Subjects made valuable suggestions with regard to the SST room. To stimulate tactile senses, they suggested the use of bean bags, a massage chair, bubble tubes and fibre optics. For gustatory senses they suggested that coffee, tea, and snacks and biscuits should be available. They suggested the use of aromatherapy to stimulate olfactory senses, and calming background music to stimulate auditory senses. To stimulate visual senses they suggested bubble tubing, laser and fibre optics, moving lighting balls, mirrors and wall units. These suggestions were taken into consideration when setting up the SST intervention.

### Demographic data and resilience scores

Subjects of all three groups were females with an average age of 43, and a standard deviation of 10.23. The minimum age of subjects was 21 and the maximum age 64. According to a *p*-value of 0.29 there was no significant difference in age.

In the pilot group, the average age of subjects was 48 with a standard deviation of 10.49. The average age of the comparison group was 44 with a standard deviation of 8.34, and the average age of the intervention group was 42 with a standard deviation of 10.91.

The health of subjects before the intervention was good overall: 21% of subjects’ health was excellent, 17% of subjects’ health was very good, 49% of subjects’ health was good and 13% of subjects’ health was fair. Results before the intervention indicated that 30% of subjects never felt depressed, whilst 6% of subjects felt depressed all the time. 29% of subjects indicated that they sometimes felt depressed and 35% frequently felt depressed.

After the completion of the resilience questionnaire Cronbach’s reliability coefficient was 0.875 with an average inter-item correlation of 0.232, indicating that the resilience questionnaire was reliable. Therefore, individual items are not discussed, but the total resilience of all three participating groups. Before the intervention the average resilience of all three participating groups was 137.83 with a standard deviation of 15.17 and a minimum of 99 and a maximum of 172. All three participating groups thus demonstrated moderate high levels of resilience. After the intervention the average resilience of all three participating groups was 142.27 with a standard deviation of 14.85 and a minimum of 96 and a maximum of 174. Thus, it reflects moderate high levels of resilience, as seen in Wagnild and Young’s (1993) differentiation of the various levels of resilience.

### Statistical significance of pre- and post-test results

#### Pretest results

According to the results of ANOVA and Cohen’s *d* coefficient there was no meaningful statistical difference with regard to the pilot, comparison and intervention group’s resilience pretest results. A *p*-value of 0.647 confirms this result (see Tables 1 and 2).

**TABLE 1:** Cronbach’s alpha reliability coefficient before intervention.

Group	Mean	Standard deviation	Cronbach alpha	Standardised alpha	Average inter-item correlation
Pilot	138.9	18.2	0.90	0.90	0.40
Intervention	137.0	14.7	0.90	0.90	0.20
Comparison	139.0	17.0	0.90	0.90	0.30
All groups	137.7	15.5	0.88	0.88	0.23

*Source:* Author’s own work

**TABLE 2:** Results of ANOVA on pretest of resilience – Cohen’s d coefficient.

Group	Mean	Standard deviation	*p*	Effect size Pilot with	Comparison with
Pilot	141.42	18.33	-	-	-
Comparison	138.98	15.98	-	0.13	-
Intervention	136.50	14.30	-	0.27	0.16
**Total**	**137.82**	**15.17**	**0.647**	**-**	**-**

*Source*: Author’s own work

A Spearman’s rank order correlation of −0.03 was used to determine whether age had an effect on resilience, which indicates that the correlation between age and resilience was not statistically or practically important.

#### Post-test results

After the intervention each group’s dependent *t*-test on increased resilience contained the following results: In the pilot group, who did not participate in the intervention, subject’s average resilience was 140.50 with a standard deviation of 15.18, a *p*-value of 0.87 and an effect size of 0.05. This indicates that there was no statistical improvement in the pilot group’s resilience over time. In the comparison group, who also did not participate in the intervention, the subjects’ average resilience was 135.86 with a standard deviation of 11.27, a *p*-value of 0.44 and a small effect size of 0.22, indicating that there was no statistical improvement in the comparison group’s resilience either. In the intervention group subjects’ average resilience was 146.36 with a standard deviation of 13.95, a *p*-value of 0.00 and a big effect size of 0.79, indicating a significant statistical increase in the intervention group’s resilience.

**FIGURE 2:**
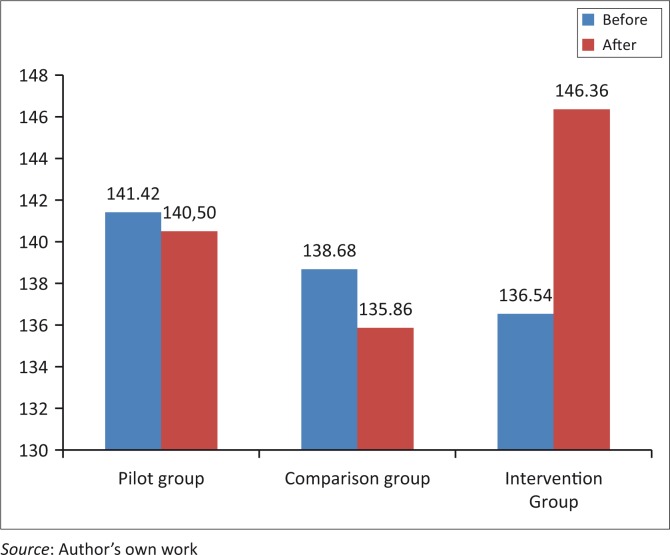
Resilience levels of different participating groups before and after sensory stimulation therapy (SST) intervention.

**TABLE 3:** Spearman’s rank order correlation.

Variable	Age	Resilience
Age	1.00	−0.03
Resilience	−0.03	1.00

*Source*: Author’s own work

**TABLE 4:** Dependent *t*-test in each group on increase in resilience.

Variable	Mean	Standard deviation	*p*	Effect size
**Group – Pilot**				
Resilience 2	140.50	15.18	-	-
Resilience	141.42	18.33	0.87	0.05
**Group – Comparison**				
Resilience 2	135.86	11.27	-	-
Resilience	138.68	12.80	0.44	0.22
**Group – Intervention**				
Resilience 2	146.36	13.95	-	-
Resilience	136.54	12.50	0.00	0.79

*Source*: Author’s own work

Results of ANOVA on post-test controlling for pretest differences confirmed there were no statistically significant differences between the participating groups.

### Narratives

The short narratives written by the subjects in the intervention group confirm that their resilience might have been strengthened through the SST intervention. Themes emerging from the narratives included that SST *created a warm*, *welcoming atmosphere*, and that subjects experienced an immediate relief of tension when utilising the SST room.

Subjects shared that SST created a welcoming atmosphere and positive experience, especially when they could spend time in the SST room and experience a change from the clinical environment of the OR to a warmer atmosphere. A subject shared the following in a narrative: *Die kere wat ek daar was het dit n mens rustig gemaak. Was n ontvlugting gewees van die werk. Het gevoel of n mens in die woud gaan stap met jou eie gedagtes*. (The times I spent there made one quiet. Was an escape from work. Felt as if one walks in the forest with your own thoughts).

*Immediate relief of tension* was experienced, namely decreased stress levels, relaxation between cases, willingness to return positively to work conditions and increased resilience were reported after visits to the SST room. For example, one subject described her experience in the SST room as ‘a total escape from all the stress and high work demands’. Another subject shared the following: *Ek kan met eerlikheid se dat die sensoriese kamer het wondere verrig vir my emosionele toestand. Dit was n kans om weer jouself by mekaar te kry en om rustig te wees en te oordink. Dit was n tyd om jouself weer te vind*. (I can say with honesty that the sensory room did wonders for my emotional condition. It was a chance to get yourself together again and to be quiet and to reflect. It was a time to find yourself again).

Subjects also commented on the SST, namely that the time available to spend in the SST room was limited because of the nature and amount of theatre procedures. Subjects reported that the equipment used, according to their specific needs and suggestions, were sufficient. Although limited space allowed only a few visitors at a time, it still complied with the requirements for an SST room.

## Ethical considerations

Ethical permission was granted to the study, which formed part of the RISE study with ethical clearance from the Ethics Committee of the North-West University (Ref. No. NWU-00036-11-A1). Furthermore, informed consent was obtained, as explained earlier.

**TABLE 5:** ANCOVA post-test controlling for pretest differences.

Group	Resilience 2 mean	Resilience 2 standard error	*p*	Effect size
Pilot	139.08	4.06	-	0.75
Comparison	135.97	3.30	-	1.02
Intervention	147.67	1.93	-	0.27
**Total**	**-**	**-**	**0.647**	**-**

*Source*: Author’s own work

Subjects benefited from the SST intervention. There was no direct benefit for the pilot and comparison groups, but they were informed of the indirect benefit that their participation contributed to the formulation of recommendations to strengthen the resilience of OR nurses.

Potential hazards were limited in the following manner: The researcher ensured anonymity by using a coding system for the questionnaires and record keeping of SST visits. Subjects visited the SST room individually or in small groups, as decided by themselves. The SST room is a private, comfortable room in the OR. They could visit the SST room as many times as they chose, for as long as they chose – within the policy of the OR.

Subjects are furthermore protected through storing data on a password-protected computer for a period of 5 years. Hard copies of the questionnaires, SST record and narratives are kept in a locked cupboard, also for a period of 5 years, after which it will be destroyed.

## Rigour

### Reliability

The Cronbach’s alpha reliability coefficients and Clark and Watson’s inter-item correlation coefficient (Clark & Watson 1995; Cronbach 1951) determined the internal consistency and reliability of the resilience questionnaire. The strength of the relationship between the variables was analysed by means of Cohen’s *d* effect size indicator before and after the intervention. Cohen’s criteria can be interpreted as *d* – 0.2 = small, *d* – 0.5 = medium and *d* – 0.8 = large (Christensen *et al.* 2011; Cohen 1988). As mentioned earlier, the Cronbach’s reliability coefficient was 0.875 with an average inter-item correlation of 0.232 in this research, indicating that the resilience questionnaire was reliable to measure the resilience of OR nurses.

### Validity

Rigour is affected by four types of validity, namely statistical validity, internal validity, construct validity and external validity (Polit & Beck 2012). Statistical validity and construct validity were ensured by using an already existing and validated resilience questionnaire. Because this was a quasi-experimental study, internal validity could not be fully ensured, but it was strengthened through competent decisions with regard to the most appropriate design and method for the study, a thorough description of the intervention and monitoring of the use of the intervention. Furthermore, the themes obtained from the narratives confirmed that it might have been possible that the intervention contributed to strengthening the resilience of the subjects.

External validity was ensured to a limited extent, taking the relatively small sample size into consideration, through being able to apply the results in similar settings, namely other OR settings in private hospitals in the North West Province. In addition, intervention validity was ensured through conducting information sessions, continuously implementing the intervention, monitoring the utilisation of the intervention and daily maintenance of the SST room.

## Discussion

The purpose of the study was to determine the effectiveness of SST as an intervention to strengthen the resilience of OR nurses in a private hospital in the North West Province. The objectives of the study were to explore and to describe OR nurses’ needs for SST, OR nurses’ suggestions with regard to the implementation of SST and to explore and to describe the effectiveness of an SST intervention in order to strengthen the resilience of OR nurses in a private hospital in the North West Province. The collected data enabled the researcher to reach these objectives and to make several conclusions.

OR nurses are generally exposed to high levels of stress in their working environment. There is also a global shortage of nurses, especially in specialised departments, such as the OR. Insufficient information about interventions to improve the resilience of nurses, especially in the OR, was available. As a trained professional theatre nurse, the researcher noticed this need to explore the resilience of nurses in the OR.

According to subjects participating in this research there was definitely a need for the implementation of an SST intervention in the OR because of a high workload, shortage of experienced nurses and high turnover rates leading to decreasing resilience levels. None of the subjects was familiar with SST and neither did they ever participate in an SST intervention. SST was a total new experience to subjects in the intervention group. Stimulation of subject’s five primary senses in a relaxed and calm atmosphere increased their resilience levels. The consequence was higher levels of hope, optimism, coping, self-efficacy and a sense of coherence – all characteristics of resilience (Koen *et al.* 2011a).

The measuring instrument used in this study was highly reliable. A resilience scale questionnaire was used to determine subject’s resilience levels before and after the implementation of the intervention. There was no statistical difference in resilience levels before the intervention between the intervention and comparison groups. However, after the intervention there was a statistically significant increase in the intervention group’s resilience. Thus, it indicated that participation in an SST intervention may strengthen the resilience of OR nurses in a private hospital in the North West Province. SST thus has the potential to strengthen the resilience of OR nurses who work in strenuous work environments and who need resilience to cope. Health service managers of operating theatres in private hospitals should consider implementing SST rooms to strengthen the resilience of OR nurses.

## Limitations of the study

The study is limited in that it comprised a relatively small sample size. The findings can therefore not be generalised to the wider population of OR nurses in general.

Furthermore, the aroma therapy as part of the SST intervention had a negative effect on some of the subjects, causing sinus irritation, and preventing them from using the SST room regularly. The researcher thus realised that a short-coming in the self-report questionnaire to determine the needs and suggestions of subjects with regard to SST was that there were no questions on possible allergies of subjects.

This was a quasi-experimental design with limited control over the intervention and the authors thus acknowledge that the intervention might not have been the only cause for the improvement in the resilience of the subjects.

## Recommendations

Recommendations for nursing practice include that an SST room can be implemented in the OR to strengthen the resilience of OR nurses. The SST room should be flexible and should be designed according to the needs of the OR nurses using the room. Any member of the multi-disciplinary team, including OR nurses, can implement the SST room. Sensitivity to aroma therapy should be determined and taken into consideration when implementing the SST room. Regular in-service training on resilience and SST should be held to keep OR nurses updated.

With regard to education and training, SST can be included in the curricula for undergraduate training as an intervention to strengthen the resilience of nurses, and regular in-service training sessions on SST and resilience can be held in private health-care settings.

## Conclusion

It can be concluded that the research objectives have been met, and that the hypothesis that participating in SST will strengthen the resilience of OR nurses in a private hospital in the North West Province is supported. The resilience of OR nurses’ who participated in the SST intervention was strengthened, as evident from the results of the resilience questionnaires and narratives. Further research should explore the implementation of SST in various departments in private hospitals. Attributes of resilience and factors influencing resilience in the private health-care sector can also be investigated.
